# Identification of epigenetic signature associated with alpha thalassemia/mental retardation X-linked syndrome

**DOI:** 10.1186/s13072-017-0118-4

**Published:** 2017-03-10

**Authors:** Laila C. Schenkel, Kristin D. Kernohan, Arran McBride, Ditta Reina, Amanda Hodge, Peter J. Ainsworth, David I. Rodenhiser, Guillaume Pare, Nathalie G. Bérubé, Cindy Skinner, Kym M. Boycott, Charles Schwartz, Bekim Sadikovic

**Affiliations:** 1 Department of Pathology and Lab Medicine, Western University, London, ON Canada; 2 Children’s Hospital of Eastern Ontario Research Institute, University of Ottawa, Ottawa, ON Canada; 3 Molecular Genetics Laboratory, Victoria Hospital, London Health Sciences Center, 800 Commissioner’s Road E, B10-104, London, ON N6A 5W9 Canada; 4 Department of Paediatrics, Western University, London, ON Canada; 5 Department of Biochemistry, Western University, London, ON Canada; 6 Department of Oncology, Western University, London, ON Canada; 7 Children’s Health Research Institute, London, ON Canada; 8 Department of Pathology and Molecular Medicine, McMaster University, Hamilton, ON Canada; 9 Department of Clinical Epidemiology and Biostatistics, McMaster University, Hamilton, ON Canada; 10 Center for Molecular Studies, J.C. Self Research Institute of Human Genetics, Greenwood Genetic Center, Greenwood, SC USA

**Keywords:** ATRX, DNA methylation, Epi-signature, Intellectual disability, Biomarker

## Abstract

**Background:**

Alpha thalassemia/mental retardation X-linked syndrome (ATR-X) is caused by a mutation at the chromatin regulator gene *ATRX*. The mechanisms involved in the ATR-X pathology are not completely understood, but may involve epigenetic modifications. ATRX has been linked to the regulation of histone H3 and DNA methylation, while mutations in the *ATRX* gene may lead to the downstream epigenetic and transcriptional effects. Elucidating the underlying epigenetic mechanisms altered in ATR-X will provide a better understanding about the pathobiology of this disease, as well as provide novel diagnostic biomarkers.

**Results:**

We performed genome-wide DNA methylation assessment of the peripheral blood samples from 18 patients with ATR-X and compared it to 210 controls. We demonstrated the evidence of a unique and highly specific DNA methylation “epi-signature” in the peripheral blood of ATRX patients, which was corroborated by targeted bisulfite sequencing experiments. Although genomically represented, differentially methylated regions showed evidence of preferential clustering in pericentromeric and telometric chromosomal regions, areas where ATRX has multiple functions related to maintenance of heterochromatin and genomic integrity.

**Conclusion:**

Most significant methylation changes in the 14 genomic loci provide a unique epigenetic signature for this syndrome that may be used as a highly sensitive and specific diagnostic biomarker to support the diagnosis of ATR-X, particularly in patients with phenotypic complexity and in patients with *ATRX* gene sequence variants of unknown significance.

**Electronic supplementary material:**

The online version of this article (doi:10.1186/s13072-017-0118-4) contains supplementary material, which is available to authorized users.

## Background

An emerging development in the field of medical genetics has been the identification of Mendelian disorders involving genes encoding the writers, erasers, readers and remodelers of the epigenetic machinery [1]. Building on several decades of evidence regarding the functions of covalent DNA methylation [2, 3] and histone modifications [4] in regulating gene transcription, it is evident that mutations in the proteins responsible for creating, interpreting or removing the broad arrays of epigenetic marks can be linked to genetic conditions including cancer [5], imprinting disorders and/or single-gene disorders [6].

Along with these discoveries came the opportunity, not only for the elucidation of underlying molecular mechanisms altered in these disorders, but also for the identification of epi-signatures that can be diagnostically useful, specifically where patients express a subset of clinical manifestations associated with a phenotypic spectrum shared across more than one syndrome, making a specific clinical diagnosis difficult to reach.

Among the rapidly expanding number of proteins responsible for chromatin maintenance and remodeling related to transcription is alpha thalassemia/mental retardation X-linked (ATRX; NG_008838.2). Mutations in the *ATRX* gene cause alpha thalassemia/mental retardation X-linked syndrome (ATR-X, OMIM 301040), a disorder characterized by moderate to severe intellectual disability, expressive language disorder, characteristic facial gestalt during infancy, often associated with hematological signs of alpha thalassemia [7].

The ATRX protein functions as an agent of ATP-dependent chromatin remodeling and is a member of the SWI/SNF superfamily of proteins. The latter can have a wide variety of cellular functions, as described in detail in several recent reviews [8–10]. Briefly, ATRX protein is involved in cellular processes such as meiosis, mitosis, DNA repair and regulation of transcription through an effect on chromatin [11–15]. Disruption of these activities may contribute to developmental abnormalities associated with the ATR-X syndrome.

Within the ATRX protein, a histone-binding ATRX–DNMT3–DNMT3L (ADD) domain can sense the methylation modifications of both H3K4 and H3K9 [16], essentially acting as an interpreter of these histone states. ATRX is also known to associate with the transcription cofactor DAXX. ATRX–DAXX complex is responsible for deposition of histone H3.3 at the telomeric and pericentromeric heterochromatic regions within chromosomes [17]. Loss of ATRX in ES cells leads to the loss of histone H3.3 at imprinting control regions and telomeric regions, along with the concurrent loss of H3K9me3 [18, 19]. ATRX has also been linked to DNA methylation regulation, as mutations at the *ATRX* gene result in DNA methylation changes at subtelomeric and repetitive regions [20].

The role of ATRX as a regulator of heterochromatin dynamics raises the possibility that mutations in *ATRX* may lead to downstream transcriptional effects across the complex of genes or repetitive regions involved in the global context of the disorder, in addition to explaining phenotypical differences in these patients. For example, *ATRX* mutations affect the expression of α-globin gene cluster, causing α-thalassemia [21]. Mechanistically, α-globin cluster, among other genes, has G-rich tandem repeats (TRs) sites, which have been reported to bind ATRX resulting in H3.3 deposition and gene expression regulation. In addition, it was suggested that the differences in size of these TRs among ATR-X patients contribute to the ranges in severity of the syndrome [22].

The orchestrated regulation of epigenetic mechanisms, including associations between ATRX and DNA methylation [11, 12], is essential for tissue homeostasis, cell identity and proper human development. Here, we describe the findings of a genome-wide DNA methylation array (GWMA) performed on peripheral blood samples from patients with ATR-X and show the genome-wide changes in DNA methylation that occur in patients with this epigenetic syndrome. We have identified a specific epi-signature of differentially hypo- and hypermethylated genes in patients clinically diagnosed with ATR-X syndrome. Our study shows the preponderance of differentially methylated genes within, or adjacent to, pericentromeric or telomeric chromosomal regions, suggesting a major role of heterochromatin in the pathophysiology of ATR-X, linked to the disruption of ATRX function in the context of its role as a regulator of heterochromatin dynamics.

## Results

### The epi-signature identified in blood samples from ATR-X patients

**Fig. 1:**
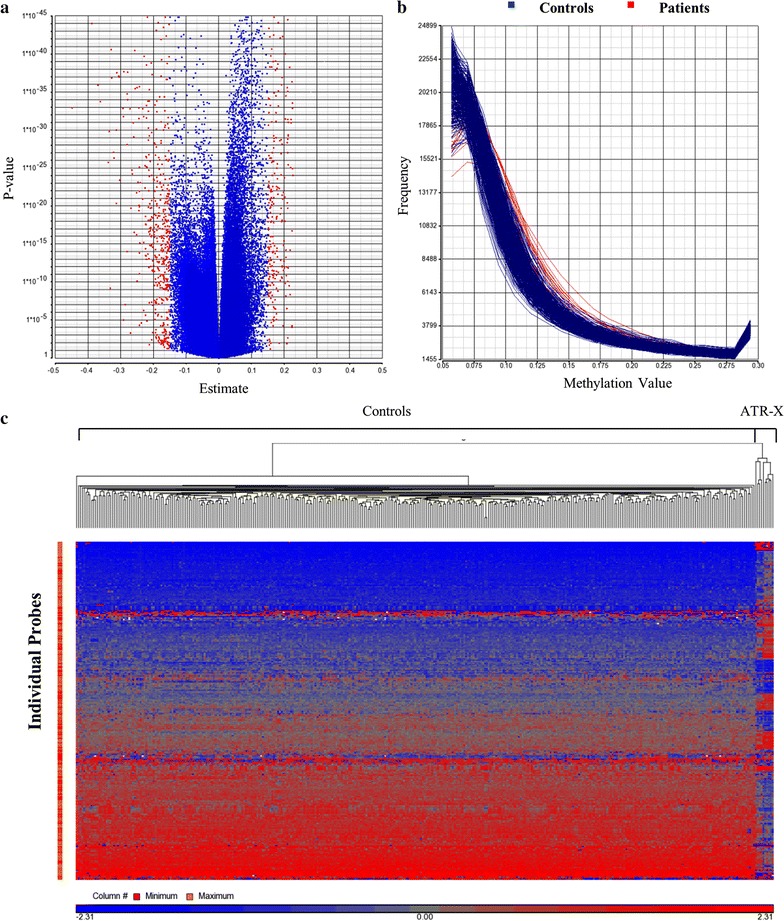
**a** Volcano plot of methylation difference (estimate) between ATR-X and controls versus statistical significance (log *p* value) of individual probes. In *red* are highlighted probes with estimate value higher than 0.15. Positive estimate value = hypermethylation; negative estimate value = hypomethylation. **b** Histogram of all samples showing methylation value (*X-axis*) versus frequency of methylation levels across the genome (*Y-axis*) in ATR-X patients (*red*) and controls (*blue*). Patients with ATR-X showed a higher frequency of methylation at low methylation value regions (0.1–0.2 methylation value). Low methylation value regions are characteristic of promoter CpG islands. **c** Hierarchical clustering of probes differentially methylated (*p* < 0.01) between ATR-X and controls demonstrating marked asymmetry of the two groups. Cases are represented in the columns and probes in the rows

The genome-wide DNA methylation array of 20 blood samples obtained from ATR-X patients was compared with a reference cohort (controls). Various methylation changes at a single-probe level were identified across the genome, consisting of both hypo- and hypermethylation (estimate value > ±0.15) (Fig. 1a). Hierarchical clustering of significant probes (*p* < 0.01) clearly demonstrated a unique methylation profile and subclustering for these patients compared with our large laboratory reference cohort (Fig. 1c). The global methylation analysis revealed an increase in methylation at low methylation value regions (0.1–0.2 methylation value) in patients relative to controls (Fig. 1b). This pattern suggests that increased methylation is taking place in normally unmethylated regions, majority of which are normally located in promoters and CpG islands.

Statistical filtering to identify regions with most robust methylation changes, using multiple parameters including *p* value <0.01, *F* value >50, number of consecutive probes >4 and methylation difference > ±20%, revealed 16 genetic regions with significant statistical difference between ATR-X and controls (Table 1). Of these, 13 regions showed hypermethylation (methylation difference higher than +0.2) and three regions showed hypomethylation (methylation difference lower than −0.2). These regions were distributed across the genome both outside (*n* = 5) and within CpG islands (*n* = 11), including seven regions at gene promoter CpG islands and two intragenic CpG islands (Table 1). This epi-signature was specific to ATR-X patients and did not correlate with the type of mutation at the *ATRX* gene locus.

**Table 1:** ATR-X methylation signature: significant regions detected by methylation array in ATR-X patients (*n* = 17) compared with controls (*n* = 210)

Chr	Region start	Region stop	# Probes	Nearest gene	Distance to gene (bp)	Within CpG island	Gene promoter/intragenic
chr1	228291476	228291715	5	*C1orf35*	453	No	–
chr3	107810351	107810801	6	*CD47*	415	Yes	–
chr3	109056339	109056907	4	*DPPA4*	0	No	Promoter
chr4	124222	125122	5	*ZNF718*	0	Yes	Intragenic/promoter
chr5	23506728	23507762	13	*PRDM9*	0	No	promoter
chr5	134073420	134073589	5	*CAMLG*	581	No	–
chr5	150284292	150284806	9	*ZNF300*	0	Yes	Promoter
chr5	150325572	150326872	13	*ZNF300P1*	0	Yes	Promoter
chr6	168045258	168045898	5	*LOC401286*	21624	No	–
chr6	34498908	34499514	5	*PACSIN1*	0	Yes	Intragenic
chr8	43131250	43132517	9	*POTEA*	15068	Yes	–
chr10	38069509	38069955	4	*ZNF248*	0	Yes	Intragenic
chr16	10479552	10480299	6	*ATF7IP2*	0	Yes	Promoter
chr19	12305543	12306507	7	*LOC100289333*	0	Yes	Promoter
chr20	623079	623431	4	*SRXN1*	3837	Yes	–
chrY	21664286	21665031	4	*BCORP1*	0	Yes	Promoter

Significant regions: Human reference genome Hg19. Probes >4, estimate (net methylation difference) > ± 20%, *F* value >50, *p* value <0.01. Gene promoters were defined as any sequence immediately surrounding the annotated 5ʹ end of the gene

We then performed a single-patient analysis to identify possible patient-specific, as opposed to patient cohort-specific, recurring methylation changes, using statistical parameters of *p* < 0.01, methylation difference > ±15%, across four consecutive probes. First we observed that the cohort-specific epi-signature was absent in one of the patients (patient #12). A follow-up assessment showed that although this patient has previously been identified to carry a possible mutation in the *ATRX* gene, more recent data demonstrated that c.5579A>G; p.N1860S in the *ATRX* gene is indeed a benign polymorphism and hence this patent did not have the ATR-X syndrome. The remaining 17 patients with molecular diagnosis of ATR-X, using the above statistical cutoffs, showed an average of 9.8 significant loci from the epi-signature per individual, with the minimum of four significant loci observed in two patients (#13 and #15). To evaluate the specificity of this assay, we applied the ATR-X epi-signature in randomly selected 15 individuals, which included normal controls that were not part of the discovery cohort, as well as individuals with Fragile X syndrome, Prader–Willi syndrome, Angelman syndrome and Beckwith–Wiedemann syndrome. The majority of these individuals did not present any statistical significant changes at the ATRX epi-signature loci. Six control individuals showed significant changes at 1 or 2 ATRX epi-signature loci (*POTEA* and *PACSIN1*). These two genes had slightly higher level of variable DNA methylation and were as a result removed from the final ATRX epi-signature.

### Biological interpretation

A more comprehensive gene list with methylation changes in the ATR-X group passing the following criteria, minimum of three consecutive probes with *p* < 0.01, *F* value across the region >50 and methylation difference > ±15% (i.e., at least a 15% methylation difference), showed an overrepresentation of genes involved in biosynthetic processes, nucleic acid metabolic processes and methylation process (Table 2). Many of the genes are involved in transcriptional regulation: *PRDM9* encodes a histone H3 lysine-4 trimethyltransferase [23]; CTDP1 functions in recruiting RNA polymerase to DNA promoters and is an OMIM gene for congenital cataracts, facial dysmorphism and neuropathy [24]; TFB2M regulates mtDNA transcription and maintenance [25]; also ZNF300, ZNF274 and ATF7IP2 are transcriptional regulators [26–28]. Another gene, *QKI*, regulates RNA splicing, export of target RNAs from the nucleus, translation of proteins and RNA stability [29]. In addition, three genes encode proteins associated with methylation process, including the histone H3 lysine-4 trimethyltransferase, a known target of ATRX and the betaine–homocysteine methyltransferase 2-BHMT2, which catalyzes the methylation of homocysteine. Evidences suggest that ATRX functions as a high-affinity RNA-binding protein and may regulate RNA stability of function [30]. These findings further support previous evidences, suggesting that ATRX has a role in regulating DNA and RNA metabolism and stability, as well as in the epigenetic regulation. Mutation at the *ATRX* gene may result in transcriptional deregulation of several genes across the genome and consequently neurodevelopmental problems associated with the disorder.

**Table 2:** Biological pathways identified by pathway analysis of the differentially methylated genes in ATR-X

Biological pathway	Biosynthetic process		Nucleic acid metabolic process		Methylation process
Number of genes in group	14		12		3
Fisher’s exact enrichment score −ln(*p* value)	5.9		4.6		4.6
Fisher’s exact right-tail *p* value	<0.01		0.01		0.01
Genes	DPPA4	PPAP2C	ZNF274	ATF7IP2	PRDM9
	ZNF718	ZNF486	CTDP1	ZNF486	BHMT2
	PRDM9	ZNF274	TFB2M	ZNF300	TFB2M
	BHMT2	TFB2M	QKI	DPPA4	
	ZNF300	ATF7IP2	ZNF718	ZNF248	
	QKI	CTDP1	UBE2U	PRDM9	
	ZNF248	ALG10B			

Pathway analysis was performed with differentially methylated regions using cutoff of estimate > ±0.15, *p* < 0.01, *F* > 50 and probes >3

### Technical validation of the methylation array

**Fig. 2:**
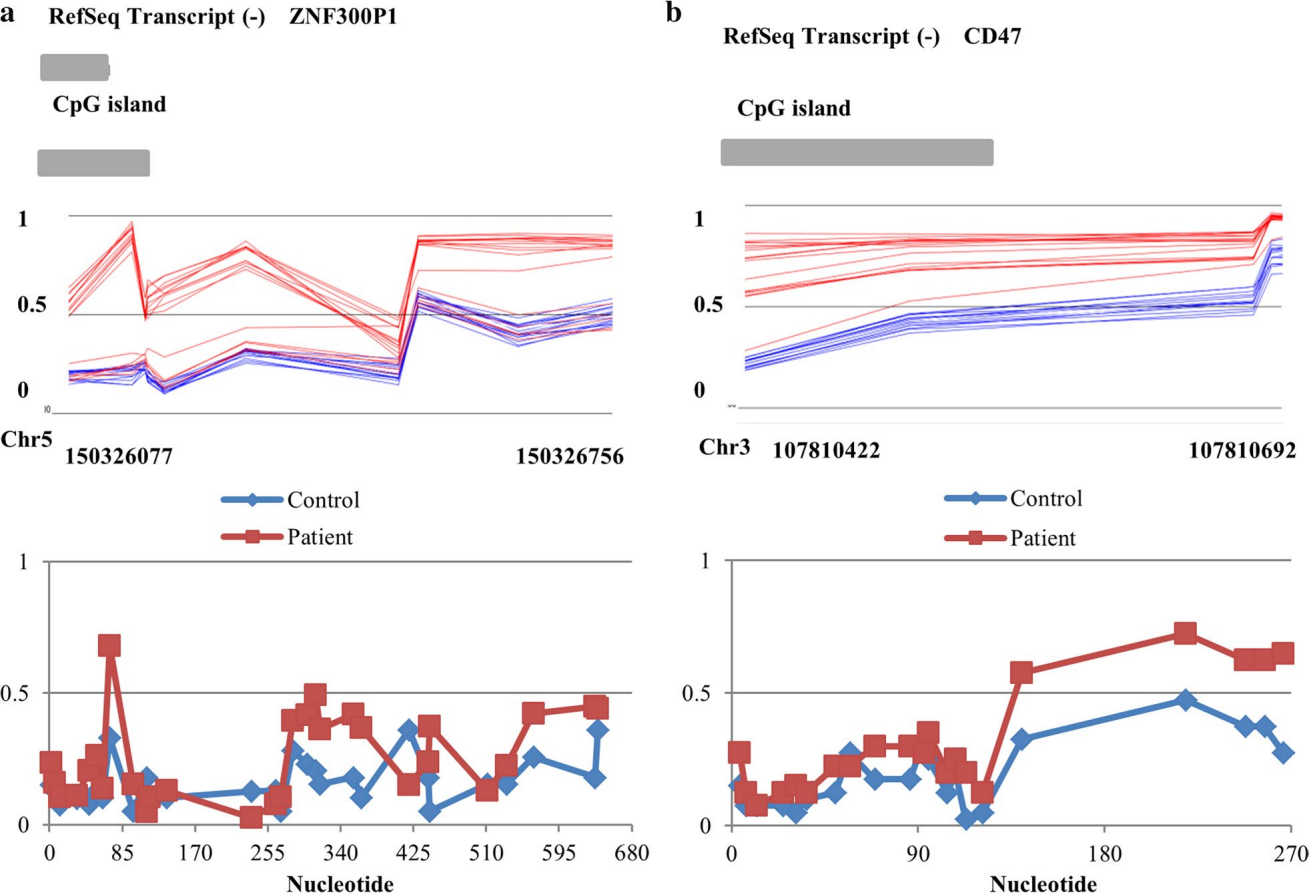
Methylation visualization of significantly altered genes *ZNF300P1* (**a**) and *CD47* (**b**) in ATR-X patients (*red*) and controls (*blue*) identified by methylation array (*top images*) and bisulfite mutagenesis sequencing (*bottom images*). *Top* and *bottom images* show the same genomic coordinates. Methylation array figures were generated using Genomic Browser viewer (Partek) and shows methylation level 0 (not methylated) to 1 (100% methylated). CpG island and gene location and chromosome coordinates are also represented. The *bottom image* corresponds to methylation average based on bisulfite sequencing data from two ATR-X patients and two controls across the same regions from the top image. Bisulfite mutagenesis and sequencing analysis of these regions confirms effects seen by methylation array analysis

To technically confirm the methylation array findings, we performed bisulfite mutagenesis/sequencing for two regions identified by the array: CD47 and ZNF300P1 (Fig. 2; Additional file 1). The methylation array at these regions showed hypermethylation in ATR-X patients compared with controls (Fig. 2), with an average methylation value for the 6 CD47 probes of 0.55 and 0.8 in controls and patients (1.45 fold increase), respectively, and average methylation for the 12 probes at ZNF300P1 of 0.3 and 0.54 in controls and patients (1.8 fold increase), respectively. Using bisulfite mutagenesis/sequencing, we detected an overall increase in methylation at these regions in patients as compared to controls (Fig. 2). The bisulfite analysis showed average methylation at CD47 gene of 0.19 and 0.31 in controls and patients (1.63 fold increase), respectively, and at ZNF300P1 gene of 0.16 and 0.25 in controls and patients (1.56 fold increase), respectively. These findings confirm the accuracy and specificity of the DNA methylation array data.

### Uneven distribution of altered methylation sites across the genome

**Fig. 3:**
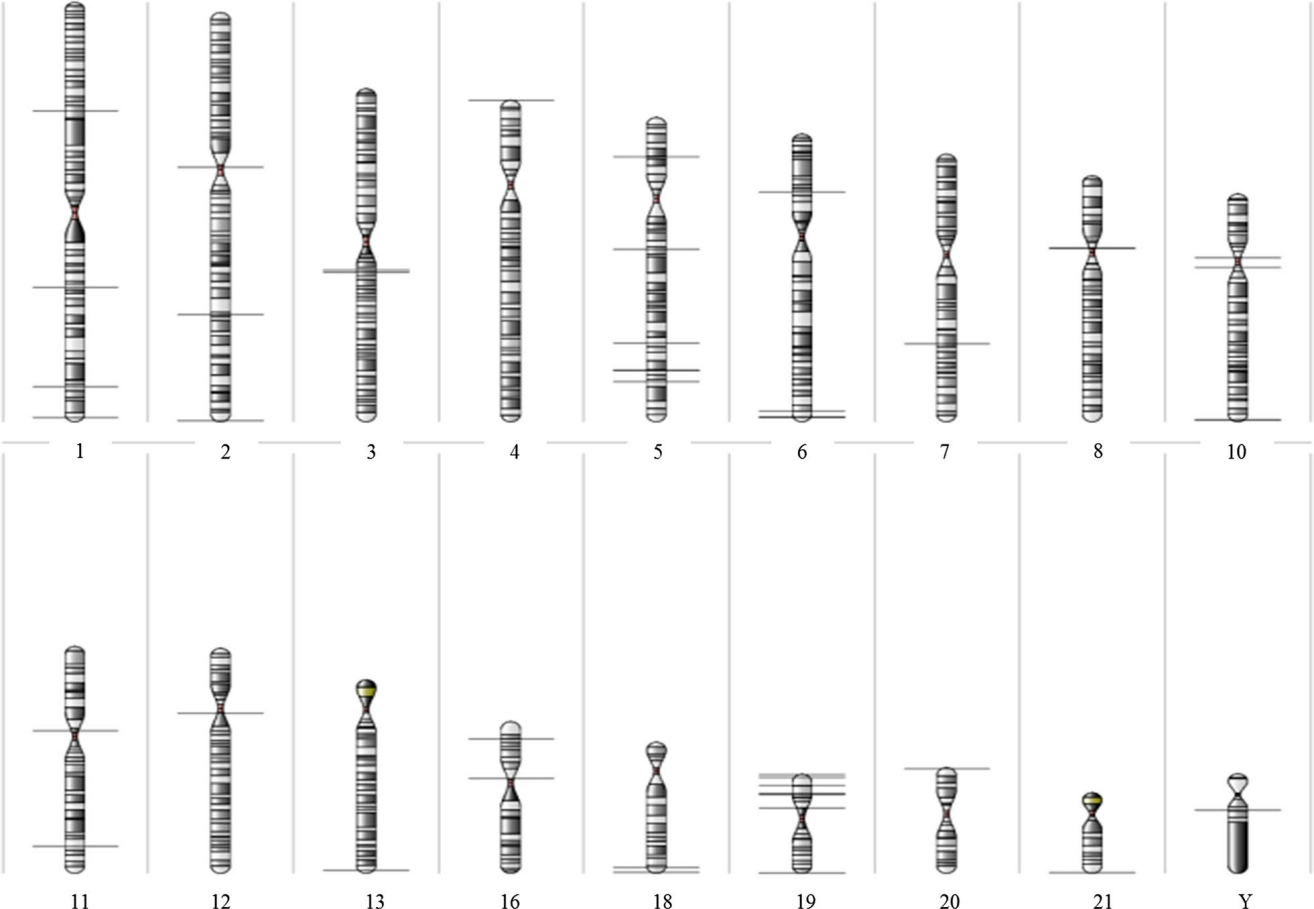
Chromosomal localization of regions with significant methylation change in ATR-X patients using a less stringent cutoff of estimate > ±0.15, *p* < 0.01, *F* > 50 and probes >3. Figure shows location of significant methylation locus (*horizontal line*) at each chromosome. Pericentromeric and telomeric regions were defined as the last full cyto-band region on the chromosome

ATRX has multiple functions related to maintenance of heterochromatin and genomic integrity that are essential during mammalian development. Hence, we hypothesized that genes in the ATRX epi-signature may be clustered in highly heterochromatinized pericentromeric and telomeric regions of the chromosomes. Using a statistical cutoff of *p* < 0.01, estimate > ±0.15, *F* value >50 and probes >3, the top 40 genes were localized using Karyogram view (Partek GS). We found that 17/40 (42.5%) of these genes in our signature mapped to telomeric and subtelomeric regions (defined as the last full cyto-band region on the chromosome) and 8/40 (20%) to pericentromeric regions (defined as the full cyto-band region on the centromere of the chromosome), with the remaining 15 (37.5%) scattered throughout the genome (Fig. 3). These findings suggest that ATRX dysfunction has a functional consequence to genes localized within heterochromatinized regions of the genome, with potentially inappropriate gene expression of these genes/epi-signature secondary to loss of ATRX as a regulator of chromatin integrity.

## Discussion

The interplay between ATRX and DNA methylation has been evidenced by early studies in EBV-transformed cells from patients with ATR-X and controls, showing that mutations at the ATRX gene cause changes in the pattern of methylation at subtelomeric and rDNA sequences [20]. Furthermore, loss of ATRX expression has been linked to extensive epigenomic alteration including CPG island hypermethylation observed in astrocytic tumors [31, 32]. The involvement of ATRX in the regulation of DNA methylation was further supported by the discovery that ATRX interacts with MeCP2 and cohesion subunits in the brain [33, 34]. MeCP2 is a methyl-CpG-binding domain protein with affinity for GC-rich sequences and methylated DNA which in turn facilitates the recruitment of histone modifiers and chromatin remodeling complexes [35]. Similar to ATRX, MeCP2 is essential for neurodevelopment and mutations or duplications of the *MeCP2* gene cause Rett syndrome, a neurodevelopmental disorder [36]. In addition, cohesin proteins play a role in the regulation of chromosome organization and gene expression by binding to unmethylated CTCF-associated regions and mutations at cohesin genes are associated with the developmental defects seen in patients with Cornelia de Lange syndrome [37]. MeCP2 was shown to recruit the helicase domain of ATRX to heterochromatic regions in a DNA methylation-dependent manner [33]. In addition, MeCP2 has been reported to interact with DNA methyltransferase 1 in order to perform maintenance methylation in vivo [38], as well as with histone H3 lysine 9 methyltransferase enzymes, to reinforce a repressive chromatin state by bridging DNA methylation and histone methylation [39].

Both ATRX protein and de novo DNA methyltransferases DNMT3A/B/L contain a histone-binding domain (ADD) that has been shown to play a role in the establishment and maintenance of DNA methylation patterns. The ADD domain interacts with specific methylation modifications of histone lysine 4 and 9 (H3K4 and H3K9). The H3K4 methylation is associated with gene transcription and promoters DNA hypomethylation, whereas methylation of H3K9 is a heterochromatin-associated mark associated with transcriptional repression and DNA hypermethylation [16, 17]. H3K4 and H3K9 methylation is proposed to act as chromatin-based signals for regulation of DNA methylation, while ATRX–heterochromatin interaction depends on these histone methylation markers [16]. ATRX ADD domain binds to the methylated H3K9 (H3K9me3) in conjunction with unmodified H3K4 which are commonly seen in the repressed repeat elements. Therefore, mutations that functionally disrupt the ATRX protein and result in “mis-targeting” of ATRX–heterochromatin interaction may provide a mechanism for abnormal DNA methylation patterns in patients with the ATR-X syndrome. While ATRX does not contain a DNA methyltransferase domain, we and others [20] have clearly shown association between ATRX mutation and abnormal patterns of DNA methylation.

The mechanism for ATRX induction of DNA methylation aberrations is not well known. Evidence has shown an overlapping function of ATRX and ATF7IP2. A genome-wide promoter DNA methylation study, using methylation-dependent immunoprecipitation–Chip assay, has demonstrated hypermethylation at ATF7IP2 gene in patients with ATRX mutation [40]. ATF7IP2, also known as MBD1-containing chromatin-associated factor 2, is known to bind to the transcription repression domain of the methylated cytosine-binding protein MBD1, as well as to interact with the H3K9 methyltransferase SETDB1 [28]. The overlapping protein interaction of ATRX–ATF7IP2 suggests that they form part of the same repressive chromatin complex, which involves ATF7IP2-induced H3K9me3 and ATRX binding to H3K9me3. There is also evidence for ATF7IP2 and ATRX transcriptional activation role, through SP1 and DAXX interaction, in promyelocytic leukemia nuclear bodies [28, 41, 42]. These data suggest that ATRX and ATF7IP2 have overlapping repressive/activating chromatin remodeling properties and potentially function in overlapping gene regulation pathways.

Most studies assessing the regulation of DNA methylation by ATRX have been focused on repetitive sequences and gene-specific methylation analysis. A recent study using methylation-sensitive restriction endonuclease has shown that *ATRX* mutations are associated with alterations in the DNA methylation profiles in highly repetitive sequences [20]. Another study using bisulfite mutagenesis analysis in mice model has demonstrated that specific gene activation at ancestral pseudoautosomal regions, which are repetitive sequences regulated by ATRX, does not involve gene-specific changes on DNA methylation, but relies on the ATRX-dependent H3.3 deposition mechanism [43]. However, none of these studies have analyzed global DNA methylation and/or specific gene CpG islands methylation in non-repetitive sequences. By using a high-resolution methylation array technique and a large reference cohort (controls), our study has clearly shown the existence of a pattern of DNA methylation changes, including in promoter CpG islands, telomeric and pericentromeric regions, in patients with ATR-X. Accordingly, in our study, most of the DNA methylation changes observed in patients with an *ATRX* gene mutation were localized at telomeric and pericentromeric regions. How the epigenetic consequences of ATRX mutations actually result in the disease phenotype is not well understood. It is possible that the methylation alterations could result in differences in transcriptional regulation. For example, hypomethylation in a gene promoter CpG island may result in increased transcription, whereas hypermethylation may result in decreased transcriptional activity [6]. Gene pathway analysis showed that many of the genes identified in the ATR-X epi-signature are associated with DNA and RNA metabolic process, which may be involved in the regulation of specific gene expression and corroborate to the cardinal developmental processes disrupted in this rare disease; however, further research involving integrative analysis of gene expression and DNA methylation profiling to investigate the relationship between these DNA methylation changes and gene expression is warranted.

Here, we propose that the most significant and recurrent regions altered in the genomic DNA of patients with ATR-X, consisting of 14 loci, provide an epigenetic signature for this syndrome which may be used as a high sensitive and specific diagnostic biomarker to support the diagnosis of ATR-X, particularly in patients with phenotypical complexity and/or with *ATRX* gene sequence variants of unknown significance. Previous findings have demonstrated evidence of loss of DNA methylation in the repetitive elements [20]. While theoretically, it would be possible to use repetitive element methylation patterns as part of a unique ATRX mutation epi-signature, routine analysis of the repetitive elements DNA methylation pattern can be challenging due to the lack of specificity for assays designed for assessment of methylation of genomic repeats. Furthermore, most array or sequencing-based bisulfate protocols are limited to targeting unique genomic sequences. Therefore, identification of a robust unique epigenetic signature across a large number of unique genetic sequences that we describe in this manuscript presents an opportunity for utilization of these findings in routine clinical diagnostics.

In addition to the ATR-X epi-signature described here, our group has recently demonstrated unique DNA methylation signatures in patients with two other conditions, including Floating–Harbor syndrome, which is caused by mutation in the *SRCAP* gene, as well as cerebellar ataxia, deafness and narcolepsy syndrome, which is caused by mutations in the *DNMT1* gene [44, 45]. Other groups have also identified epi-signatures in patients with Sotos syndrome [46], and the X-linked intellectual disability caused by the *KDM5C* gene [47]. Taken together, these studies demonstrate the ability of genome-wide methylation array to accurately diagnose multiple epigenetic disorders. Utilization of this technology in routine clinical practice will enable the discovery of new epigenetic biomarkers and will serve to enhance our understanding of human disease etiology. However, the identification of epigenetic variants of unknown clinical significance (E-VUS) will require delivery of testing to be performed in regulated clinical laboratories along with an adequate control cohort of normal samples, together with the development and implementation of clinical and laboratory testing guidelines, and availability and integration with pre- and posttest genetic counseling.

## Conclusion

In conclusion, the observation of genome-wide epigenetic defects in ATR-X patients expands our understanding of the pathology of this disease, in which specific DNA methylation changes could lead directly to an aberrant expression of a number of genes in *ATRX*-deficient patients, particularly, but not restricted to telomeric and pericentromeric regions, thus contributing to the phenotypes associated with ATR-X syndrome. In addition, the unique epi-signature identified for ATR-X syndrome can now be used as an epigenetic biomarker to support the diagnosis of new patients using a sensitive, specific and cost-effective GWMA testing protocol.

## Methods

### Sample collection, DNA extraction and genotyping

Peripheral blood samples from patients referred for genetic testing at the Greenwood Genetic Center were collected for methylation study. All patients were consented and counseled for ATRX testing as part of their clinical referral. Ethical approval was consented by the Self Regional Healthcare Institutional Review Board (IRB #26). Genomic DNA was extracted from peripheral blood leukocyte using standard techniques. Patients presenting with the alpha thalassemia/mental retardation X-linked syndrome (ATR-X) underwent molecular diagnostic confirmation (ATRX gene analysis) and were selected for the methylation study. Table 3 shows the molecular (mutations) characteristics of these patients. The ATR-X panel of patients is composed of 18 males with average age of 12.2 years (ranging from 8 m to 27 years).

**Table 3:** Clinical and molecular characteristics of ATR-X patients referred for methylation study

ATR-X patient no.	Mutation
1	c.109C>T; p.R37X
2	c.109C>T; p.R37X
3	c.109C>T; p.R37X
4	c.109C>T; p.R37X
5	c.109C>T; p.R37X
6	c.730A>C; p.I244L
7	c.758T>C; p.L253S
8	c.736T>C; p.R246C
9	c.736C>T; P.R246C
10	c.952G>T; p.G249C
11	c.4817G>A; p.S1606N
12	c.5579A>G; p.N1860S
13	c.5786A>G; p.K1929R
14	c.6254G>A; p.R2085H
15	c.6593A>G; p.H2198R
16	c.7156C>T; p.R2386X
17	c.7366_7367 InsA; p.M2456Nfs X42
18	Deletion of exon 2

The methylation array of these patients was compared with a reference cohort composed of controls and individuals previously referred for microarray with no significant methylation alteration. The reference cohort (controls) is composed of 210 male controls with average age of 7.3 years (2 m–53 years).

### Methylation array and data analysis

The DNA methylation array was performed using the Infinium HumanMethylation450 BeadChip (Illumina) according to standard protocol at the Genetic and Molecular Epidemiology Laboratory at McMaster University. The array coverage includes >485,000 individual methylation sites, 99% of RefSeq genes and 96% of CpG islands. Beta and intensity values for methylation were generated using the Illumina Genome Studio Software, and .idat files were imported to Partek Genomic Suite software (Partek GS). The patient cohort was compared with the laboratory reference cohort. Statistical analysis was performed to compare ATR-X patients versus the control cohort using the ANOVA test to generate probe-level statistics, including *p* value (*t* test), *F* value (signal to noise) and estimate value (net methylation difference). The cutoff of estimate > ±0.20, *p* < 0.01, *F* > 50 and probes >4 was used to select the top significant regions to be included in the epi-signature. A less stringent cutoff of estimate > ±0.15, *p* < 0.01, *F* > 50 and probes > 3 was used for pathway analysis and karyogram view in order to include a larger number of regions in those analysis. Significant regions were mapped against the CpG islands and gene promoter regions. Genomic visualization of the data was performed using the karyogram view toll (Partek GS) for chromosome distribution of differentially methylated regions, and the Genomic Browser Wizard (Partek GS) for locus-specific methylation levels.

### Pathway analysis

The top 45 differentially methylated genes identified using a less stringent cutoffs (Additional file 2) were assessed using the pathway analysis tool in the Partek Genomics Suite software. Briefly, statistical analysis included Fisher’s exact test and was restricted to functional groups at least two genes. Results show the enrichment *p* value (*p* value of the Fisher exact test reflective of the number of the genes in versus not in the list or functional group) and the enrichment score (negative log of the enrichment *p* value; a high score indicates that the genes in the functional group are overrepresented in the gene list).

### Bisulfite mutagenesis

Genomic DNA isolated from blood of ATR-X patients (*n* = 2) and controls (*n* = 2) was sodium bisulfite treated using the EZ DNA Methylation-Direct Kit (Zymo Research) according to manufacturer’s instructions. DNA was amplified by nested PCR and the resulting products ligated into the pGEM-T Easy vector using a TOPO-TA cloning kit (Invitrogen). Positive clones were sequenced with Applied Biosystem 3730xl DNA Analyzer technology (Center for Applied Genomics, McGill University). Clones were accepted at ≥95% conversion. Non-converted cytosine residues and mismatched base pairs were used to ensure all clones originated from unique template DNA.

## Additional files

**Additional file 1: Figure S1.** Methylation string diagrams of significantly altered regions in ATR-X patients and controls. Bisulfite mutagenesis and sequencing analysis was performed in approximately 20 alleles from each sample, and individual alleles are represented as a string of CpGs. The total average methylation for each sample is indicated. Unmethylated CpGs are represented as empty circles, and methylated CpGs as filled circles.

**Additional file 2: Table S1.** ATR-X methylation; significant regions detected by methylation array in ATR-X patients (n = 17) compared with controls (n = 210) using cutoff of probes >3, estimate >15%, F value >50, p value <0.01.

## Abbreviations

ATRX: alpha thalassemia/mental retardation X-linked protein
ATR-X: alpha thalassemia/mental retardation X-linked syndrome
DAXX: death domain-associated protein
DNMT: DNA methyltransferase
ES cells: embryonic stem cells
E-VUS: epigenetic variants of unknown clinical significance
GWMA: genome-wide DNA methylation array
H3K4: histone 3 lysine 4
H3K9: histone 3 lysine 9
KDM5C: lysine (K)-specific demethylase 5C
MeCP2: methyl CpG-binding protein 2
Partek GS: Partek Genomic Suite software
PCR: polymerase chain reaction
SRCAP: Snf2-related CREBBP activator protein
SWI/SNF: SWItch/sucrose non-fermentable family
TR: tandem repeats
