# Supplementary figures and images for: Identification of epigenetic signature associated with alpha thalassemia/mental retardation X-linked syndrome
Source: Epigenetics Chromatin. 2017 Mar 10;10:10. doi: 10.1186/s13072-017-0118-4 (PMC5345252; doi:10.1186/s13072-017-0118-4)

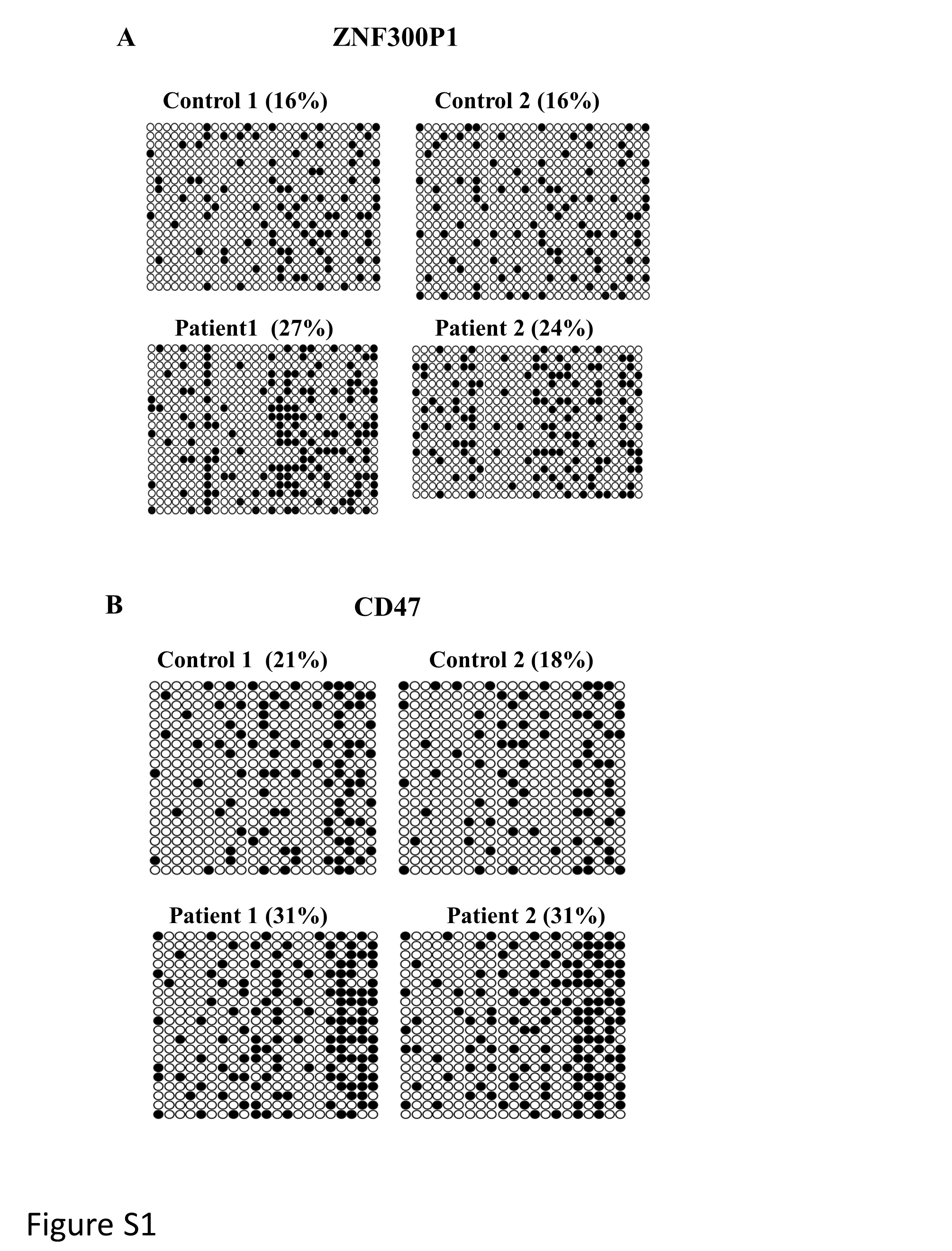

Supplement: Supplementary file 1 — Additional file 1: Figure S1. Methylation string diagrams of significantly altered regions in ATR-X patients and controls. Bisulfite mutagenesis and sequencing analysis was performed in approximately 20 alleles from each sample, and individual alleles are represented as a string of CpGs. The total average methylation for each sample is indicated. Unmethylated CpGs are represented as empty circles, and methylated CpGs as filled circles. [file 13072_2017_118_MOESM1_ESM.tif]
