# Supplementary material for: Identification of epigenetic signature associated with alpha thalassemia/mental retardation X-linked syndrome
Source: Epigenetics Chromatin. 2017 Mar 10;10:10. doi: 10.1186/s13072-017-0118-4 (PMC5345252; doi:10.1186/s13072-017-0118-4)
Supplement: Supplementary file 2 — Additional file 2: Table S1. ATR-X methylation; significant regions detected by methylation array in ATR-X patients (n = 17) compared with controls (n = 210) using cutoff of probes >3, estimate >15%, F value >50, p value <0.01. [file 13072_2017_118_MOESM2_ESM.pdf]

Table S1: ATR-X methylation: Significant regions detected by methylation array in ATR-X patients (n=17) compared with controls (n=210) using cut-off of 0.15 methylation difference

Chr	# Probes	Estimate	F value	*p*-value	Within CpG island	Nearest gene	Distance to nearest gene (bp)
chr1	3	0.178	118.3	0.000045	No	*CCDC181*	0
chr1	5	0.228	107.7	0.000045	No	*C1orf35*	453
chr2	4	-0.186	66.9	0.000045	Yes	*GGT8P*	30163
chr2	3	-0.154	54.0	0.000045	No	*TTN-AS1*	0
chr2	3	0.162	94.7	0.000045	Yes	*BOK-AS1*	3287
chr3	6	0.271	405.3	0.000045	Yes	*CD47*	415
chr3	4	0.271	187.5	0.000045	No	*DPPA4*	0
chr4	5	0.217	65.7	0.000045	Yes	*ZNF718*	0
chr5	13	-0.225	89.6	0.000045	No	*PRDM9*	0
chr5	7	0.197	106.0	0.000045	Yes	*BHMT2*	0
chr5	5	0.231	581.8	0.000045	No	*CAMLG*	581
chr5	9	0.335	303.4	0.000045	Yes	*ZNF300*	0
chr5	3	0.197	184.5	0.000045	Yes	*ADAM19*	0
chr6	6	-0.179	63.6	0.000045	No	*QKI*	507222
chr6	5	-0.295	145.1	0.000045	No	*LOC401286*	21624
chr7	3	-0.271	105.5	0.000045	No	*GPR85*	0
chr8	3	0.236	203.7	0.000045	No	*POTEA*	11130
chr10	4	0.246	149.3	0.000045	Yes	*ZNF248*	0
chr10	3	-0.300	232.2	0.000045	No	*RASGEF1A*	0
chr10	3	-0.189	60.0	0.000045	No	*INPP5A*	18901
chr11	11	0.153	82.3	0.000045	Yes	*MIR4492*	0
chr12	6	0.163	165.7	0.000045	Yes	*ALG10B*	177834
chr13	3	-0.196	61.2	0.000045	No	*ATP11AUN*	26813
chr16	3	-0.176	72.9	0.000045	Yes	*LINC00273*	2269
chr18	5	0.198	98.1	0.000045	Yes	*LOC100131655*	7094
chr19	6	0.175	210.8	0.000045	Yes	*PPAP2C*	0
chr19	4	0.156	74.8	0.000045	Yes	*MOB3A*	0
chr19	3	0.260	137.8	0.000045	Yes	*C3*	0
chr19	5	0.157	64.8	0.000045	Yes	*RGL3*	0
chr19	7	0.222	221.6	0.000045	Yes	*LOC100289333*	0
chr19	8	0.198	385.7	0.000045	No	*ZNF486*	0
chr19	8	0.159	63.8	0.000045	Yes	*ZNF274*	0
chr20	4	0.266	154.5	0.000045	Yes	*SRXN1*	3837
chr21	3	0.288	64.7	0.000045	Yes	*SPATC1L*	0
chrY	4	0.271	61.5	0.000045	Yes	*BCORP1*	0
chr11	7	0.172	83.3	0.000089	Yes	*LOC441601*	0

chr10	4	-0.185	80.7	0.000134	No	*STK32C*	0
chr8	9	0.273	124.2	0.000223	Yes	*POTEA*	15068
chr6	5	-0.205	91.6	0.000268	Yes	*PACSIN1*	0
chr6	6	0.192	51.6	0.000357	Yes	*KIF25*	0
chr1	4	0.155	67.0	0.000669	Yes	*TFB2M*	0
chr16	6	0.212	406.6	0.001115	Yes	*ATF7IP2*	0
chr5	13	0.233	237.0	0.002274	Yes	*ZNF300P1*	0
chr1	10	-0.151	115.9	0.002720	No	*UBE2U*	0
chr18	5	-0.163	57.4	0.002720	Yes	*CTDP1*	62202

Significant regions: Probes>3, Estimate > 15%, F value>50, p-value<0.01.
